# Overexpression of *GINS4* is associated with poor prognosis and survival in glioma patients

**DOI:** 10.1186/s10020-021-00378-0

**Published:** 2021-09-23

**Authors:** Binfeng Liu, Zhendong Liu, Yanbiao Wang, Xiaoyu Lian, Zhibin Han, Xingbo Cheng, Yongjie Zhu, Runze Liu, Yaoye Zhao, Yanzheng Gao

**Affiliations:** 1 Zhengzhou University People’s Hospital, Henan Provincial People’s Hospital, Henan, 450003 Zhengzhou China; 2 Department of Surgery of Spine and Spinal Cord, Henan Provincial People’s Hospital, Henan International Joint Laboratory of Intelligentized Orthopedics Innovation and Transformation, Henan Key Laboratory for Intelligent Precision Orthopedics, People’s Hospital of Zhengzhou University, People’s Hospital of Henan University, Henan, 450003 Zhengzhou China; 3 Department of Orthopedics, First Affiliated Hospital of Xinxiang Medical College, Xinjiang, China; 4 Department of Neurosurgery of the First Affiliate Hospital of Harbin Medical University, Harbin, China; 5 Department of Surgery of Spine and Spinal Cord, Henan University People’s Hospital, Henan Provincial People’s Hospital, Henan, 450003 Zhengzhou China

**Keywords:** *GINS4*, Glioma, Biomarker, Survival, Bioinformatics

## Abstract

**Background:**

*GINS4*, an indispensable component of the GINS complex, is vital for a variety of cancer. However, no known empirical research has focused on exploring relationships between *GINS4* and glioma. Thus, this study aims to understand and explain the role of *GINS4* in glioma.

**Method:**

First, we used the data in the CGGA, TCGA, GEO, GEPIA, and HPA databases to explore the expression level of *GINS4* in glioma, the correlation between *GINS4* expression and the clinical features of glioma, its impact on the survival of glioma patients, and verified the analysis results through RT-qPCR, IHC, and meta-analysis. Subsequently, GSEA enrichment analysis is used to find the potential molecular mechanism of *GINS4* to promote the malignant process of glioma and the anti-glioma drugs that may target *GINS4* screened by CMap analysis. Moreover, we further explored the influence of the *GINS4* expression on the immune microenvironment of glioma patients through the TIMER database.

**Results:**

Our results suggested that *GINS4* was elevated in glioma, and the overexpression of *GINS4* was connected with a vast number of clinical features. The next, *GINS4* as an independent prognostic factor, which can result in an unfavorable prognosis of glioma. Once more, *GINS4* may be participating in the oncogenesis of glioma through JAK-STAT signaling pathways, etc. 6-thioguanine, Doxazosin, and Emetine had potential value in the clinical application of drugs targeting *GINS4*. Finally, the expression exhibited a close relationship with some immune cells, especially Dendritic cells.

**Conclusion:**

*GINS4* is an independent prognostic factor that led to a poor prognosis of glioma. The present study revealed the probable underlying molecular mechanisms of *GINS4* in glioma and provided a potential target for improving the prognosis of glioma.

**Supplementary Information:**

The online version contains supplementary material available at 10.1186/s10020-021-00378-0.

## Background

Glioma is the most common primary intracranial malignancy, accounting for 81% of malignant brain tumors (Ostrom et al. 2014). The total age-adjusted incidence rate of glioma (adjusted by the national population of the study) varies from 4.67 to 5.73 per 100,000 people. Although relatively rare, they still cause significant mortality (Larjavaara et al. 2007; Gousias et al. 2009). According to the current medical standards, the standard treatment to reduce the mortality of gliomas is surgical combined with radiotherapy and chemotherapy. With the continuous improvement of the medical level, new treatment methods such as photodynamic therapy, immunotherapy, gene therapy, and so on have been proposed(Bush et al. 2017). Although there are many treatments for glioma, the prognosis has not been significantly improved, which is related to the difficulty of surgical resection, rapid disease progression, and high recurrence rate.

In recent years, with the development of molecular pathology, several molecular markers have been confirmed to be associated with glioma, which acts a vital guiding role in the clinical treatment of glioma, such as epidermal growth factor receptor (EGFR), O-6-methylguanine-DNA methyltransferase (MGMT), tumor protein p53 (TP53), Isocitrate dehydrogenase (IDH mutation) and 1p/19q codeletion (Melin and Jenkins 2013; Gupta and Salunke 2012; Jones et al. 2013; Riemenschneider et al. 2010). More importantly, these molecular markers have been used in the clinical treatment of gliomas and in predicting the survival and prognosis of patients. For instance, IDH mutation and 1p/19q deletion can lead to unfavorable prognosis in glioma patients (Jones et al. 2013). The lower the expression of MGMT in glioma patients, the better the chemotherapy effect of temozolomide (Gupta and Salunke 2012). Although there are currently some biomarkers used in the clinical treatment of glioma, the tumorigenesis of glioma is a complex pathological process involving multiple molecules. Thus, it is crucial to identify more molecular markers for the early identification of patients at risk and the treatment of glioma.

In yeast and Xenopus egg extracts, the formation of the GINS complex is crucial for initiating DNA replication. The yeast heterotetramer GINS complex is composed of *GINS1, GINS2, GINS3,* and *GINS4* (Takayama et al. 2003). *GINS4*, also known as *SLD5*, is an indispensable component of the *GINS* complex and plays a significant role in the initiation and extension of DNA replication (Joshi et al. 2016). DNA replication is an important step in tumor cell proliferation, so *GINS4* might acts a key role in tumor development. A growing body of evidence now suggests that *GINS4* is a key to the development of cancer. For instance, *GINS4* can promote the growth and progress of gastric cancer cells by activating *Rac1* and *CDC42* (Zhu et al. 2019). At the same time, *GINS4* can promote the progression of lung cancer by promoting the key characteristics of tumor potential and lead to poor prognosis of lung cancer patients (Yang et al. 2019). Moreover, the relationship between *GINS4* and breast cancer, colorectal cancer, hepatocellular carcinoma, and other tumors has also been confirmed (Sagredo et al. 2018; Rong et al. 2020; Lian et al. 2018). Although there are many studies have shown that *GINS4* plays an important role in tumors, the exact biological function and molecular mechanism of *GINS4* in the carcinogenesis of glioma are still unknown.

In this study, the biological function of *GINS4* in glioma and its relationship with prognosis has been first studied. For all we know, our finding presents the first evidence that *GINS4* plays an important role in the carcinogenesis of glioma, providing a potential therapeutic target for the treatment of glioma and providing new hope for improving the survival and prognosis of glioma patients.

## Materials and methods

### Data collection

GEPIA2 (http://gepia2.cancer-pku.cn/) was used to analyze the expression of *GINS4* in various tumors, there were 163 cases of glioblastoma multiforme (GBMs), 518 cases of low-grade gliomas (LGGs), and 207 normal brain tissues. We downloaded 1018 glioma RNA-seq and 301 glioma gene microarray data from the Chinese glioma genome atlas (CGGA http://www.cgga.org.cn/) database. Microarray is performed with the prior knowledge about the sequences, and RNA sequencing is performed without the prior knowledge about sequences (Chen et al. 2017). The tissue samples of CGGA RNA-seq and CGGA microarray data from different patients do not contain duplicate individuals. So, we contained the data of these two datasets samples to explore the expression of *GINS4* in gliomas, which can improve the reliability of the analysis with more tissue samples. After removing some gene data without complete clinical information, the remaining 748 RNA-seq and 268 gene microarray data were used to analyze the expression lever of *GINS4* in gliomas. At the same time, we further screened 653 glioma patients with complete clinical information from the cancer genome atlas (TCGA, https://portal.gdc.cancer.gov/) database, and analyzed the expression level of *GINS4* in glioma. The immunohistochemical (IHC) results of glioma tissues and normal brain tissues were obtained from The Human Protein Atlas (HPA, https://www.proteinatlas.org/) database for analysis of the protein expression level of *GINS4*. GSE43378, GSE4412, GSE74187, and GSE83300 data sets were obtained from Gene Expression Omnibus (GEO, https://www.ncbi.nlm.nih.gov/geo/), and these data sets are used to verify the relationship between the expression level of *GINS4* and the survival prognosis of patients with glioma.

### Cell culture

Human glioma cell lines (T98, A172, U251) and normal control cells (human astrocyte, HA) were provided by ProCell Life Science & Technology Co., Ltd (Wuhan, China). All the above cell lines were cultured in DMEM (HyClone) medium containing 10% fetal bovine serum (Gibco) and 1% penicillin–streptomycin at 37 ℃ in air containing 5% CO_2_.

### Human specimens

A total of 31 glioma tissues and 16 corresponding normal brain tissues (epilepsy was treated surgically) were obtained from patients who underwent surgical resection at Henan provincial people’s hospital, Henan, China. On the one hand, some tissue samples were further embedded in paraffin to detect the protein expression of *GINS4* by immunohistochemistry (Rong et al. 2020) glioma tissues and 6 normal brain tissues). On the other hand, the remaining tissue is immediately frozen in liquid nitrogen after tissue resection and then detect the mRNA expression level by RT-qPCR (Dam et al. 2018) glioma tissues and 10 normal brain tissues). All the study protocols were approved by the Ethics Committee of Zhengzhou University. The patients included in this study signed the informed consent according to the Declaration of Helsinki.

### RNA extraction and RT-qPCR

The total RNA of the above cell line was extracted by Total RNA Kit I (Omega Biotek). Extract the total RNA of the tissue sample by using Tri®-Reagent (Sigma, USA). After that, the concentration of RNA was detected by a NanoDrop One spectrophotometer (Thermo Fisher Scientific, USA). Finally, NovoScript Plus All-in-one 1st Strand cDNA Synthesis SuperMix (Novoprotein) and 2 × RealStar Green Fast Mixture with ROX (GenStar, A303-05) were used for total RNA reverse transcription and RT-qPCR, respectively. *GAPDH* as a normalization for *GINS4*. The primer sequences for RT-qPCR were shown in Additional file 1: Table S1.

### Immunochemical staining

The 7 high grade glioma tissues, 7 low grade glioma tissues, and 6 normal brain tissues were collected used for Immunochemical (IHC) staining. After deparaffinization in xylene and hydration in graded alcohols, the antigen of paraffin sections was retrieval by microwave oven for 15 min (EDTA, pH 8.0). Subsequently, 100ul of primary antibody against *GINS4* (GeneTex, GTX119779, 1:100 dilution) was dropped on the sections and incubated overnight at 4 °C. The horseradish peroxidase-labeled monoclonal antibody (PV6000, Zhongshan Jinqiao Biotechnology, China) was incubated at 37 °C for 40 min. Finally, the staining results were visualized by light microscopy. The IHC results were analyzed using ImageProPlus software (version 6.0).

### Gene functional analysis

Gene set enrichment analysis (GSEA) is an analysis method for whole-genome expression profile microarray data. By analyzing gene expression profile data, we can know their expression status in specific functional gene sets and whether there is some statistical significance. After downloading CGGA RNA-seq and TCGA RNA-seq data from CGGA and TCGA, we divided the data into a high expression group and a low expression group according to the expression level of *GINS4*. Then, the enrichment analysis was carried out with GSEA 4.0.jar software, and “KEGG cell signaling pathways” were selected as the gene set database for single gene GSEA. nominal (NOM) p-value < 0.05 and false discovery rate (FDR) Q-value < 0.25 were selected by permutation test (1000 times permutation test).

### Co-expression analysis

Using Pearson correlation analysis conduct the co-expression analysis of *GINS4* (Dam et al. 2018). Ten genes that are positively and ten negatively correlated with *GINS4* expression were selected based on the P-value and the correlation coefficient value. After that, we knocked down the expression of *GINS4* by small interfering RNA (siRNA) and then verified the expression level changes of *GINS4* related co-expressed genes by RT-qPCR. Sequences of all primers used are outlined in Additional file 1: Table S1.

### Cell transfection

The siRNA to *GINS4* was purchased from GenePharma (Shanghai, China). The siRNA sequence used in the study were indicated in Table 1. For transfection, cells were seeded in 6-well plates, 150 pmol siRNA was transfected into each well using Lipofectamine 2000 reagent (Invitrogen; Thermo Fisher Scientific, Inc.). After 48 h of transfection, the efficiency of transfection was detected by RT-qPCR. Then, the most efficient siRNA was selected for further experiments.

**Table 1:** RNA oligo sequence of siRNA

Gene	sequence (5′–3′)
siRNA-NC (sense)	UUCUCCGAACGUGUCACGUTT
siRNA-NC (antisense)	ACGUGACACGUUCGGAGAATT
siRNA-1 (sense)	GCCUCUCGCCGGAAGAGUUTT
siRNA-1 (antisense)	AACUCUUCCGGCGAGAGGCTT
siRNA-2 (sense)	GGACCUCUUUCGGGCAGUUTT
siRNA-2 (antisense)	AACUGCCCGAAAGAGGUCCTT
siRNA-3 (sense)	GCAGAGGGACUACGUGAUUTT
siRNA-3 (antisense)	AAUCACGUAGUCCCUCUGCTT

### CMap database screening potential drugs for glioma treatment

The key target genes related to *GINS4* co-expression obtained from CGGA RNA-seq data were input into CMap in the form of query signature file (https://pubchem.ncbi.nlm.nih.gov/) Database, by comparing the key target genes related to *GINS4* co-expression with the reference gene expression profiles in CMap, the related small molecule compounds or drugs were obtained. The results were sorted according to the score size, and the negative correlation small molecule compounds were screened out according to p < 0.01 and enrichment < − 0.8.

### Meta-analysis

We found there were no studies that report the correlation between *GINS4* and the overall survival (OS) after systematically searching by two dependent researchers (LBF and WYB) in Pubmed, Embase, Web of Science, and Cochrane Library databases. Since this is the first study to explore the relationship between *GINS4* expression and OS of Glioma. Thus, we use R software (v.4.0.0 version) to conduct the Meta-analysis to verify the prognosis value of *GINS4* in glioma patients based on CGGA, TCGA, and GEO databases. Evaluate the association of *GINS4* expression and clinical outcomes of glioma patients by using a hazard ratio (HR) with a 95% confidence interval (CI). Cochran’s Q test and Higgins I-squared (I^2^) statistics were used to examining the heterogeneity of data. If the studies contained no or moderate heterogeneity (I^2^ < 50%), a fixed-effect model was used; otherwise, the random-effects model was applied. Since there were less than 10 studies in this meta-analysis, so the Publication bias is considered reliable. p-value < 0.05 was considered to be statistically significant.

### Immune gene correlation analysis

TIMER (https://cistrome.shinyapps.io/timer/) is a database that uses RNA-Seq expression profile data to systematically analyze the immune infiltration of different types of cancer. A previously published statistical method was used to infer the abundance of tumor-infiltrating immune cells (TIICs) from gene expression profiles (Yamaguchi and Condeelis 2007). We analyzed the correlation between the expression of *GINS4* and the degree of immune infiltration through the gene modules in this database, including B cells, CD4+ T cells, CD8+ T cells, Neutrophils, Macrophages, and Dendritic cells. In addition, the Correlation between *GINS4* and immunosuppressive genes (CD274, PDCD1, and PDCD1LG2) was explored through relevant data analysis.

### Statistical analysis

All data were analyzed using R (v.4.0.0 version). A Mann-Whitney test, Chi-square or Fisher’s exact test was performed to analyze the different expression of *GINS4* between two groups using the Graphpad Prism 9.0 software. The relationship between the expression of *GINS4* and overall survival time was analyzed by Cox regression and Kaplan Meier survival analysis. Wilcox or Kruskal test was used to detect the relationship between clinical information and the expression of *GINS4* in glioma patients. Finally, the Pearson correlation coefficient was calculated to obtain the co-expression genes related to *GINS4*. The critical value of p ≤ 0.05 was statistically significant.

## Result

### Population characteristics of glioma patients

A total of 1016 CGGA data (748 CGGA RNA-seq data and 268 CGGA microarray data) and 653 TCGA RNA-seq data were obtained from CGGA and TCGA databases, respectively. The clinical information of these glioma patient gene data including age, gender, and glioma grade. In addition, postoperative radiotherapy and chemotherapy, 1p19 codeletion, and IDH mutation information were also included. The specific information is shown in Additional files 2, 3, 4: Tables S2, S3 and S4.

### GINS4 levels are increased in glioma

**Fig. 1:**
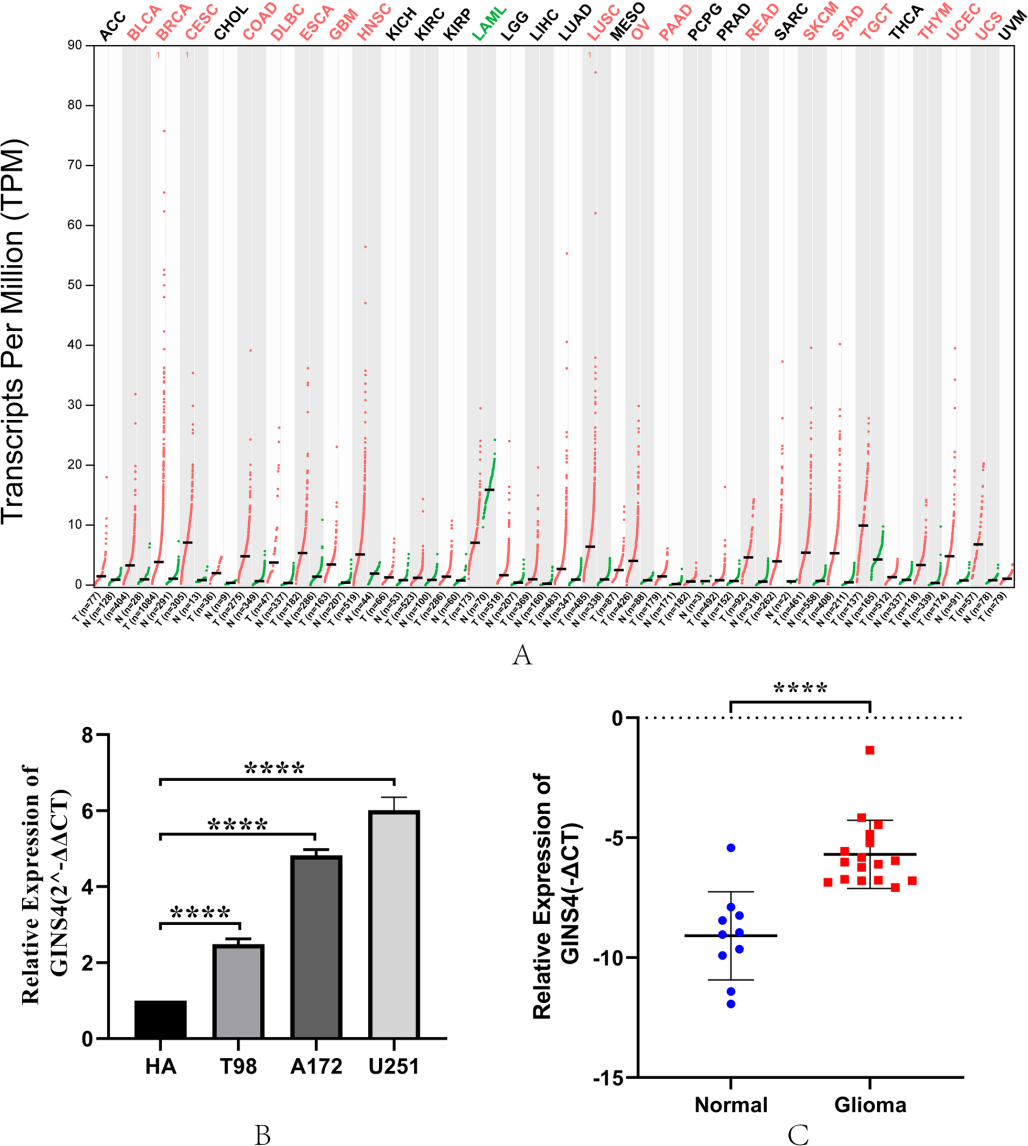
The *GINS4* was frequently upregulated in glioma. **A** Based on GEPIA2 database, the expression levels of *GINS4* in various tumors were shown. On top labels, the color of the tumor name represents the level of *GINS4* in the tumor compared with the corresponding normal tissues. Red indicates the high level of *GINS4* in tumor tissue: Bladder Urothelial Carcinoma (BLCA), Breast invasive carcinoma (BRCA), Cervical squamous cell carcinoma and endocervical adenocarcinoma (CESC), Colon adenocarcinoma (COAD), Lymphoid Neoplasm Diffuse Large B-cell Lymphoma (DLBC), Esophageal carcinoma (ESCA), Glioblastoma multiforme (GBM), Head and Neck squamous cell carcinoma (HNSC), Lung squamous cell carcinoma (LUSC), Ovarian serous cystadenocarcinoma (OV), Pancreatic adenocarcinoma (PAAD), Rectum adenocarcinoma (READ), Skin Cutaneous Melanoma (SKCM), Stomach adenocarcinoma (STAD), Testicular Germ Cell Tumors (TGCT), Thymoma (THYM), Uterine Corpus Endometrial Carcinoma (UCEC), and Uterine Carcinosarcoma (UCS); Green indicates the low level of *GINS4* in tumor tissue: Acute Myeloid Leukemia (LAML); Black indicates that there is no significant difference between the level of tumor tissue and normal tissue: Adrenocortical carcinoma (ACC), Cholangio carcinoma (CHOL), Kidney Chromophobe (KICH), Kidney renal clear cell carcinoma (KIRC), Kidney renal papillary cell carcinoma (KIRP), Brain Lower Grade Glioma (LGG), Liver hepatocellular carcinoma (LIHC), Lung adenocarcinoma (LUAD), Mesothelioma (MESO), Pheochromocytoma and Paraganglioma (PCPG), Prostate adenocarcinoma (PRAD), Sarcoma (SARC), Thyroid carcinoma (THCA), Uveal Melanoma (UVM); **B** The expression level of *GINS4* was compared between glioma cell lines and corresponding normal cell lines by RT-qPCR. **C** The expression level of *GINS4* was compared between 17 glioma tissues and 10 corresponding normal brain tissues by RT-qPCR

**Fig. 2:**
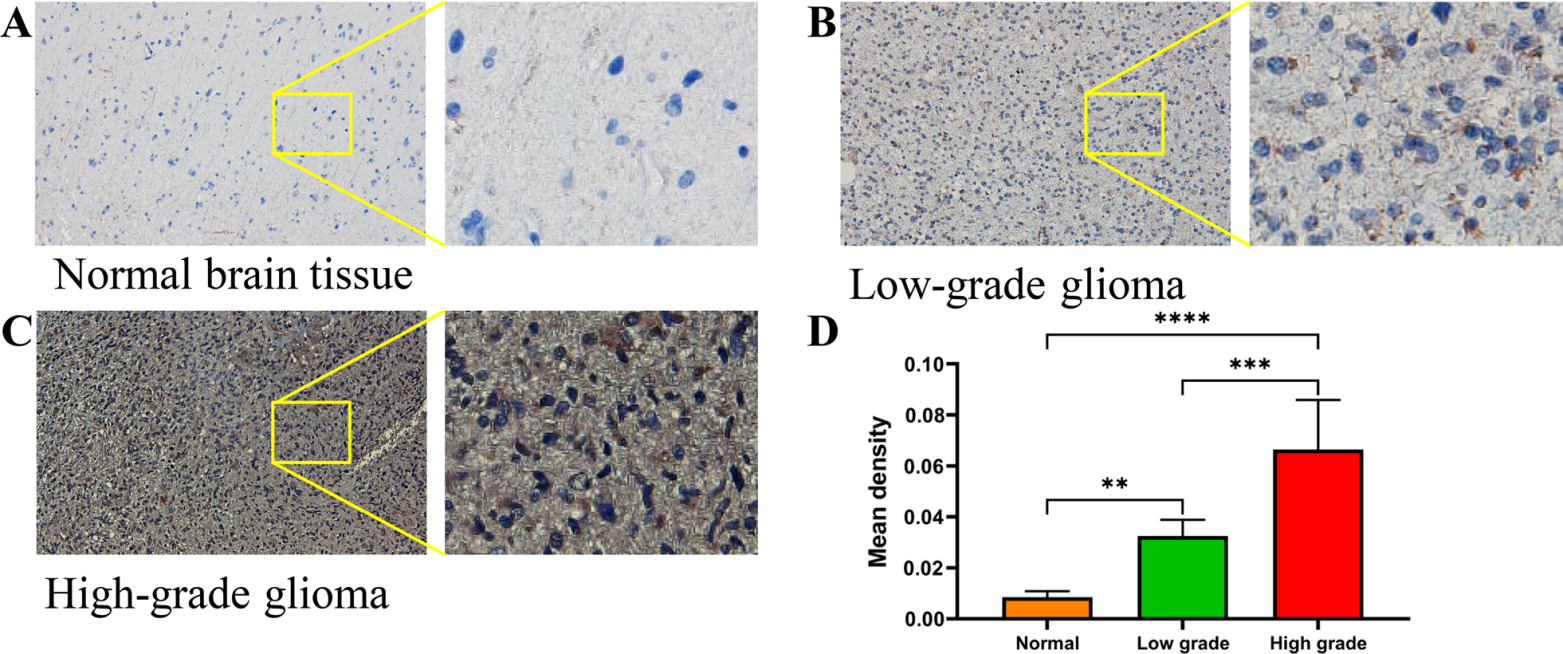
The protein expression of *GINS4* was obviously increased in glioma. **A** The staining of *GINS4* in normal brain tissue; **B** lightly positive staining of *GINS4* in low grade glioma; **C** strongly positive staining of *GINS4* in high grade glioma; **D** the statistical analysis of *GINS4* expression in glioma and control samples. *p < 0.05, **p < 0.01, ***p < 0.001, and ****p < 0.0001

First, we used GEPIA2 (http://gepia2.cancer-pku.cn/) to analyzed the *GINS4* expression in tumor tissues, and our results showed that the expression level of *GINS4* was increased in a variety of tumors, including glioblastoma (GBM) compared with those in their corresponding NT tissues (Fig. 1A). Then, we further validated that *GINS4* RNA levels were upregulated in glioma cells (A172, T98, and U251) compared with correspondence normal cell (Fig. 1B). At the same time, we investigated the expression of *GINS4* in 17 cases of gliomas and 10 cases of normal brain tissues. The results suggested that *GINS4* was elevated in gliomas compared with the corresponding normal brain tissues (Fig. 1C). Furthermore, IHC results of 2 normal brain tissues, 2 low-grade gliomas, and 2 high-grade gliomas were obtained from the HPA database. The outcome was suggesting that the level of *GINS4* protein increased in glioma tissue compared with the corresponding normal brain tissue (Additional file 5: Fig. S1). Consistent with the analysis results from HPA, our IHC staining result of 20 samples (7 high grade glioma tissues, 7 low grade glioma tissues, and 6 normal brain tissues) also verified the protein expression of *GINS4* was significantly increased in glioma, and the level of *GINS4* protein was positively correlated with the grade of glioma (Fig. 2). Collectively, these results revealed that *GINS4* is consistently upregulated in both glioma cells and tissue compared with corresponding normal cells and tissue.

### Correlation between GINS4 and clinical features of glioma

**Fig. 3:**
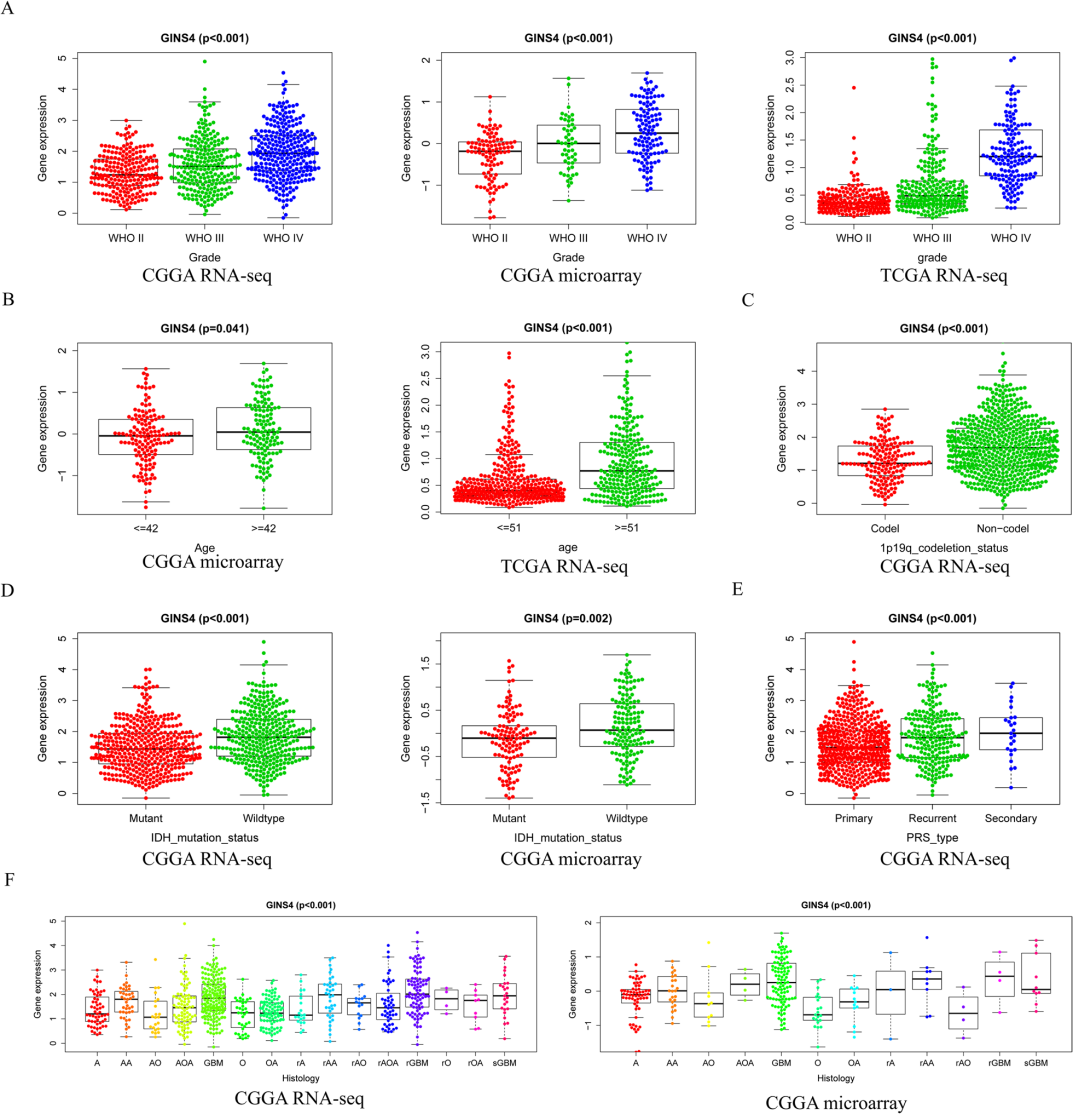
Relationship between *GINS4* expression level and multiple clinical features in the three cohorts of CGGA RNA-seq, CGGA microarray, and TCGA RNA-seq. **A** The level of *GINS4* in different grades of gliomas; **B** the relevance between the level of *GINS4* and the age of glioma patients; **C** the relationship between the level of *GINS4* and 1p/19q codeletion status; **D** the relationship between the level of *GINS4* and IDH_mutation_status; **E** the levels of *GINS4* in primary, recurrent and secondary gliomas. **F** the level of *GINS4* in gliomas of different histological subtypes

To clarify the correlation between the overexpression of *GINS4* and the clinical, pathological, and molecular characteristics. We analyze the relationship between the *GINS4* expression level and the WHO grade, age, 1p19_codeletion_status, IDH_mutation_status, PRS_type, and Histology in glioma patients by using the data of CGGA RNA-seq, CGGA microarray data, and TCGA RNA-seq. As shown in Fig. 3A, the expression level of *GINS4* was correlated with the WHO grade of glioma, and as the pathological grade of glioma increase, the average expression level of *GINS4* increase. The data of CGGA and TCGA are divided into different age groups according to the median age of these datasets when exploring the relationship between *GINS4* expression and age. Since the CGGA and TCGA databases include different populations, so the age cut-off for the two databases was different. The age cut-off for CGGA and TCGA databases are 42 and 51, respectively. The results of CGGA and TCGA databases both indicated that the expression of *GINS4* also increased with the age of glioma patients (Fig. 3B). 1p19 codeletion and IDH mutation are recognized molecular markers for the survival and prognosis of gliomas (Louis et al. 2016). Our finding revealed that the expression of *GINS4* was higher in patients without 1p19 codeletion and in patients with wild-type IDH (Fig. 3C and D). Moreover, the expression of *GINS4* in recurrent gliomas was higher than that in primary gliomas (Fig. 3E) and was also higher in several histological subtypes (Fig. 3F). Overall, these results indicate that the expression of *GINS4* is correlated with various malignancies of glioma. Therefore, we further speculate that a high level of *GINS4* in gliomas may be associated with an unfavorable prognosis of glioma patients.

### GINS4 high level indicated a poor prognosis for glioma patients

**Fig. 4:**
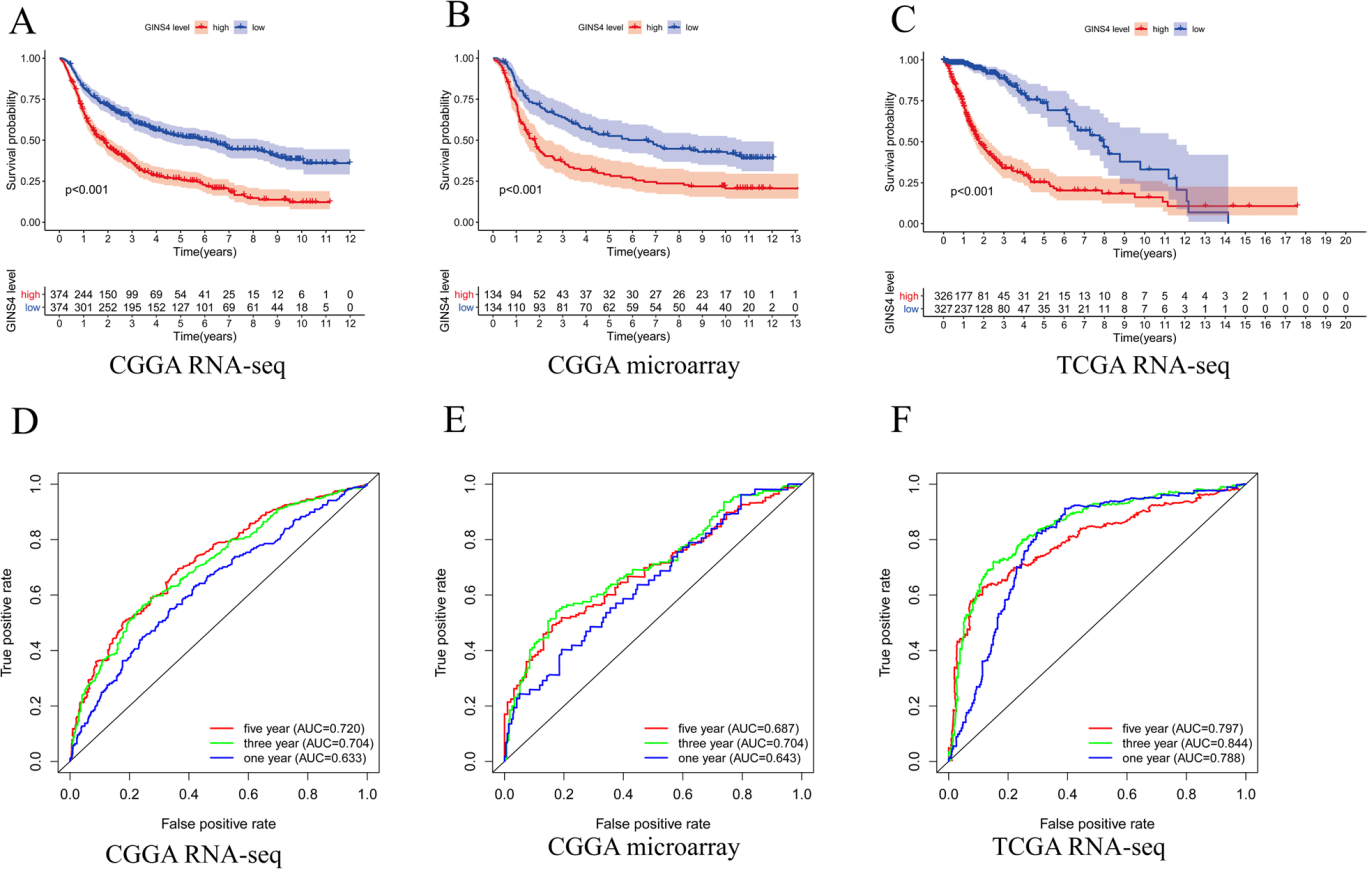
The relationship between the level of *GINS4* and overall survival of glioma patients. **A**–**C** The effect of the expression level of *GINS4* on overall survival based on the CGGA RNA-seq, CGGA microarray, and TCGA RNA-seq datasets; **D**–**F** ROC curves based on CGGA RNA-seq, CGGA microarray, and TCGA RNA-seq datasets

**Fig. 5:**
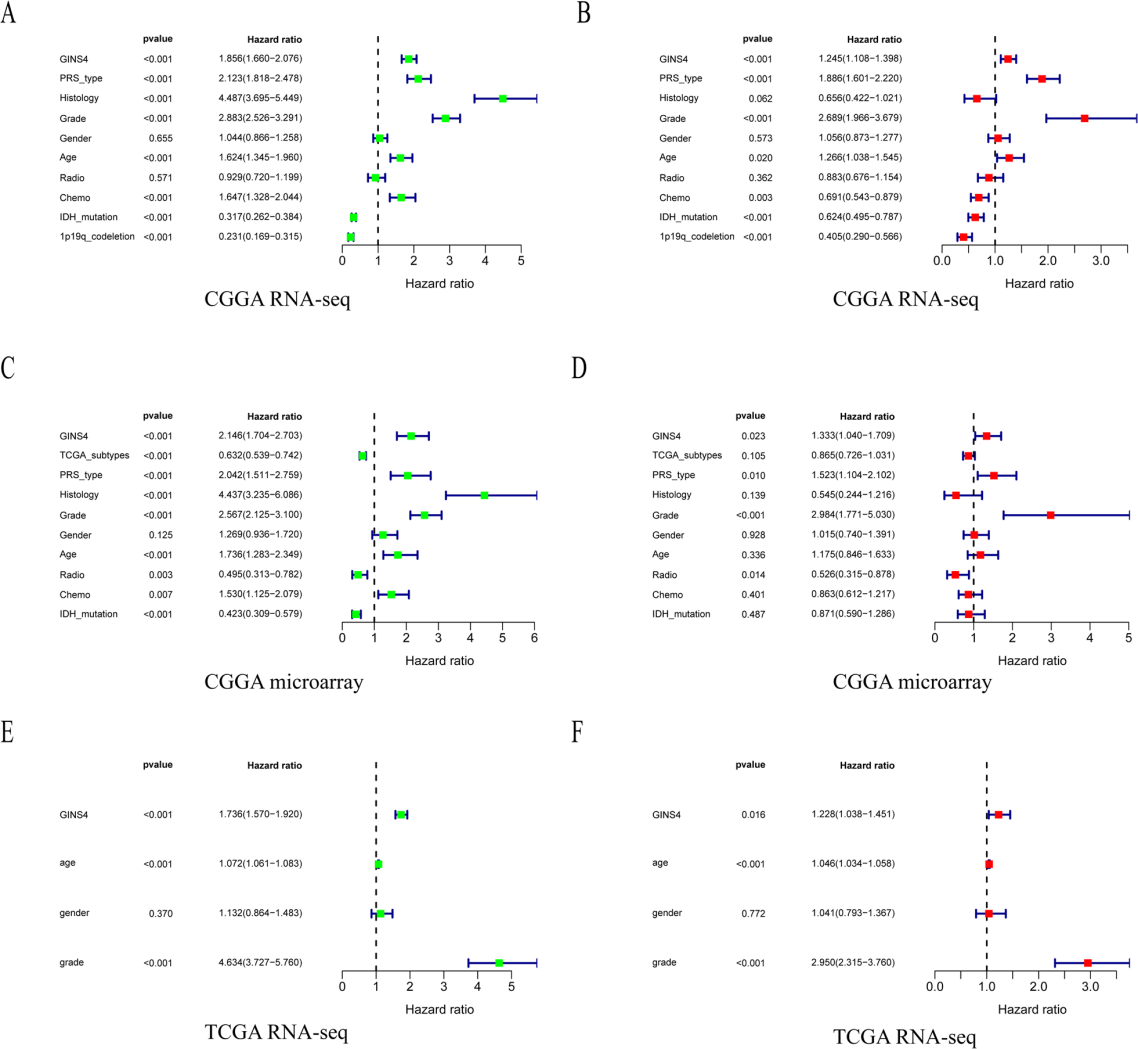
*GINS4* was an independent prognostic factor for glioma patients. **A**, **C** and **E** are univariate Cox analysis results base on CGGA RNA-seq, CGGA microarray, and TCGA RNA-seq datasets, respectively; **B**, **D**, and **F** are multivariate Cox analysis results base on CGGA RNA-seq, CGGA microarray, and TCGA RNA-seq datasets, respectively

**Fig. 6:**
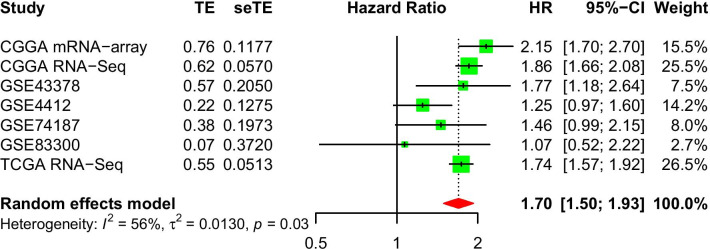
Forest plot for the relationship between *GINS4* expression and OS in glioma patients

To further explore whether our above speculation is correct, we used CGGA RNA-seq, CGGA microarray data, and TCGA RNA-seq data obtained from CGGA and TCGA databases to conduct Kaplan–Meier survival analysis. The result of survival analysis represented that *GINS4* expression in gliomas is negatively associated with the survival rate of gliomas. Patients with *GINS4* overexpression have a shorter overall survival time, and the results are consistent in the CGGA and TCGA databases (Fig. 4A–C. p < 0.001). Then, we conducted the receiver operating characteristic (ROC) curve based on the CGGA and TCGA data set to explore the diagnosis value of *GINS4* in glioma according to the area under the ROC curve (AUC). All of the above receiver operating characteristic curves demonstrate that *GINS4* had a high diagnostic value for the survival prognosis of glioma (Fig. 4D–F). What’s more, univariate and multivariate analyses were performed to explore whether *GINS4* is an independent prognostic factor affecting the prognosis of glioma. The univariate (Fig. 5A, C and E) and multivariate analyses (Fig. 5B, D, an F) results revealed that the upregulation of *GINS4* could be used as a prognostic factor for glioma. Finally, to further verify the affection of *GINS4* on the survival of glioma patients. We performed a meta-analysis to verify the diagnosis role of *GINS4* in glioma patients. Since no previous studies have reported the association between the overexpression of *GINS4* and OS in glioma patients, we only included results from seven different data sets in the meta-analysis. As shown in the Fig. 6, the pooled Hazard Ratio (HR) are 1.70 (95% CI 1.50–1.93) with moderate heterogeneity (I^2^ = 56%, p = 0.03) among the 7 datasets. Hence, we can confidently conclude that *GINS4*, as a prognostic factor, its upregulation can significantly affect the prognosis of patients with glioma.

### GSEA analysis results

**Fig. 7:**
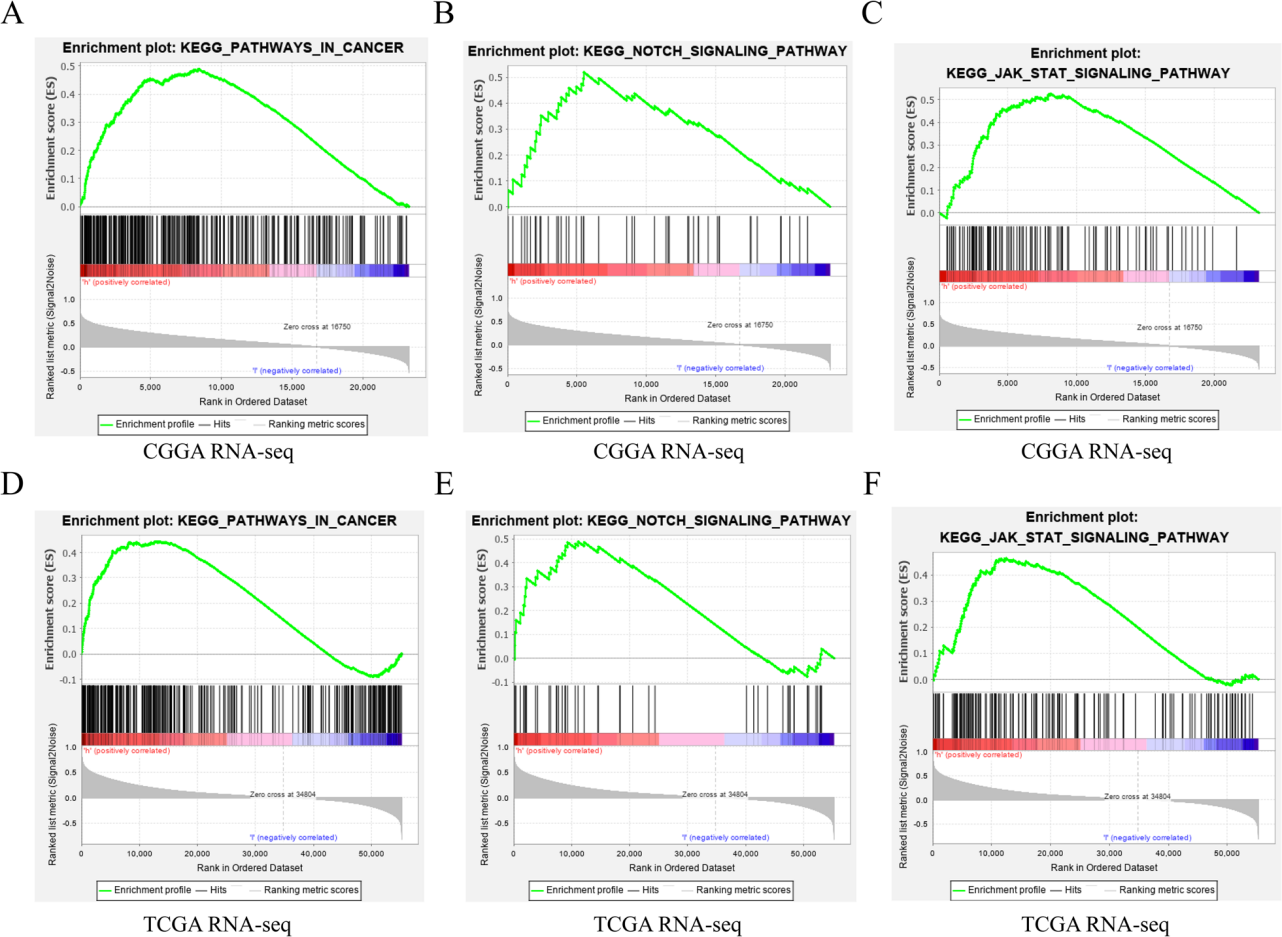
The GSEA analysis results of the CGGA RNA-seq and TCGA RNA-seq data. **A** Pathway in cancer(CGGA RNA-seq); **B** Notch signaling pathway(CGGA RNA-seq); **C** JAK-STAT signaling pathway(CGGA RNA-seq); **D** Pathway in cancer(TCGA RNA-seq); **E** Notch signaling pathway(TCGA RNA-seq); **F** JAK-STAT signaling pathwaypathway in cancer(TCGA RNA-seq)

From the above results, we can know that *GINS4*, as a carcinogene, plays a central role in the tumorigenesis of glioma, but the specific molecular mechanism that leads to the poor prognosis of glioma is still unclear. To reveal the mechanism of *GINS4* in gliomas, we used GSEA to evaluate the potential signaling pathway of *GINS4* in gliomas. We found some signaling pathways that may be related to the effect of *GINS4* on glioma progression, including pathway in cancer, NOTCH signaling pathway, and JAK-STAT pathway (Fig. 7A–F, Table 2). On the one hand, this result further illustrates the complexity of the mechanism of *GINS4* in glioma, and on the other hand, it also clarifies the potential biological pathway regulation mechanism that *GINS4* may participate in the regulation of glioma.

**Table 2:** The gene set enriches the high GINS4 expression phenotype

Gene set name	CGGA RNA-seq			TCGA RNA-seq		
	NES	NOM p-val	FDR q-val	NES	NOM p-val	FDR q-val
pathway in cancer	1.8251	0.0019	0.2210	1.8365	0.0000	0.0227
notch signaling pathway	1.6285	0.0162	0.1197	1.5887	0.0478	0.0648
jak stat signaling pathway	1.6092	0.0446	0.1253	1.6944	0.0142	0.0440

Gene sets with NOM p-value < 0.05 and FDR q-value < 0.25 were considered as significantly enriched

*NES* normalized enrichment score; *NOM* nominal; *FDR* false discovery rate

### Co-expression genes related to glioma

**Fig. 8:**
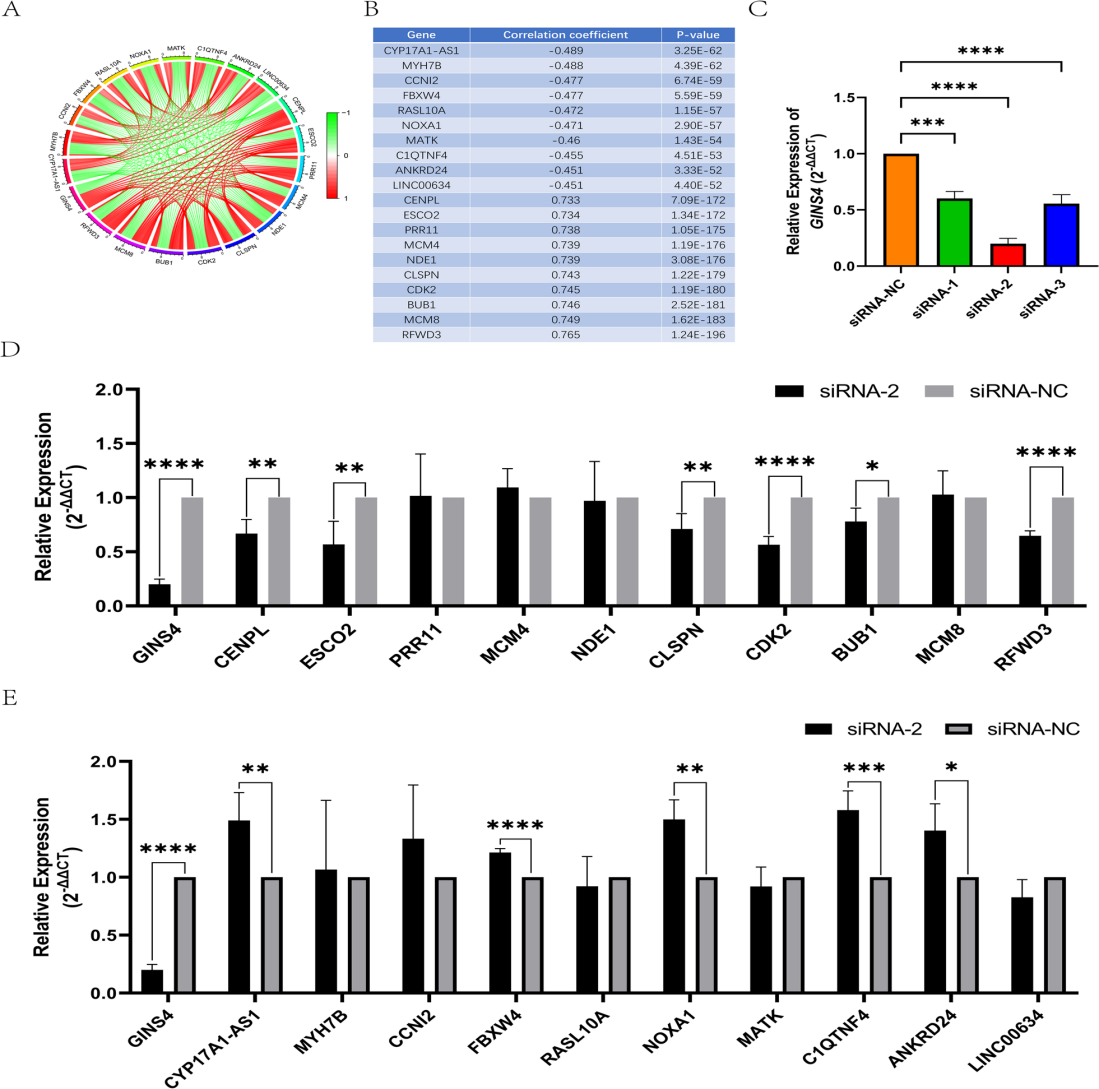
Co-expression networks show the top 10 genes that are positively and negatively correlated with *GINS4* expression in glioma patients. **A** Circle plot shows 10 positive and 10 negative genes that are most significantly co-expressed with *GINS4*; **B** Enrichment parameters of the 20 most significant genes co-expressed with *GINS4*. **C** Transfection efficiency comparison of different siRNA sequences for targeted knockdown of *GINS4* in U251 cell line; **D** The expression levels diversification of ten genes that are positively correlated with *GINS4* after the knockdown of *GINS4*; **E** The expression levels diversification of ten genes that are negatively correlated with *GINS4* after the knockdown of *GINS4*. *p < 0.05, **p < 0.01, ***p < 0.001, and ****p < 0.0001

The tumorigenesis of glioma is a complex biological process, and *GINS4* is not the only one involved in the malignant process of glioma. Therefore, we performed co-expression analysis to find more potential oncogene and tumor suppressor genes associated with glioma. Figure 8A, B were showed the top 10 positively correlated genes and the top 10 negative correlation genes that are correlated with *GINS4* expression. Subsequently, we conducted a verification test to verify the analysis results. After transfection, the expression of *GINS4* was significantly decreased in U251, and siRNA-3 had the most transfection efficiency (Fig. 8C). Moreover, our experiment results indicated that the expression of *GINS4* positive related genes(*CENPL, ESCO2, CLSPN, CDK2, BUB1, and RFWD3*) were significantly decreased, and the expression of *GINS4* negatively related genes (*CYP17A1-AS1, FBXW4, NOXA1, C1QTNF4, and ANKRD24*) was significantly elevated after the knockdown of *GINS4,* which was consistent with the prior analysis results(Fig. 8D, E). However, other related genes were not altered significantly. Collectively, those analyses and verify results broadened our horizon on the molecular regulation of GINS4 in the complex pathological process of glioma.

### Potential drugs for glioma patient

**Fig. 9:**
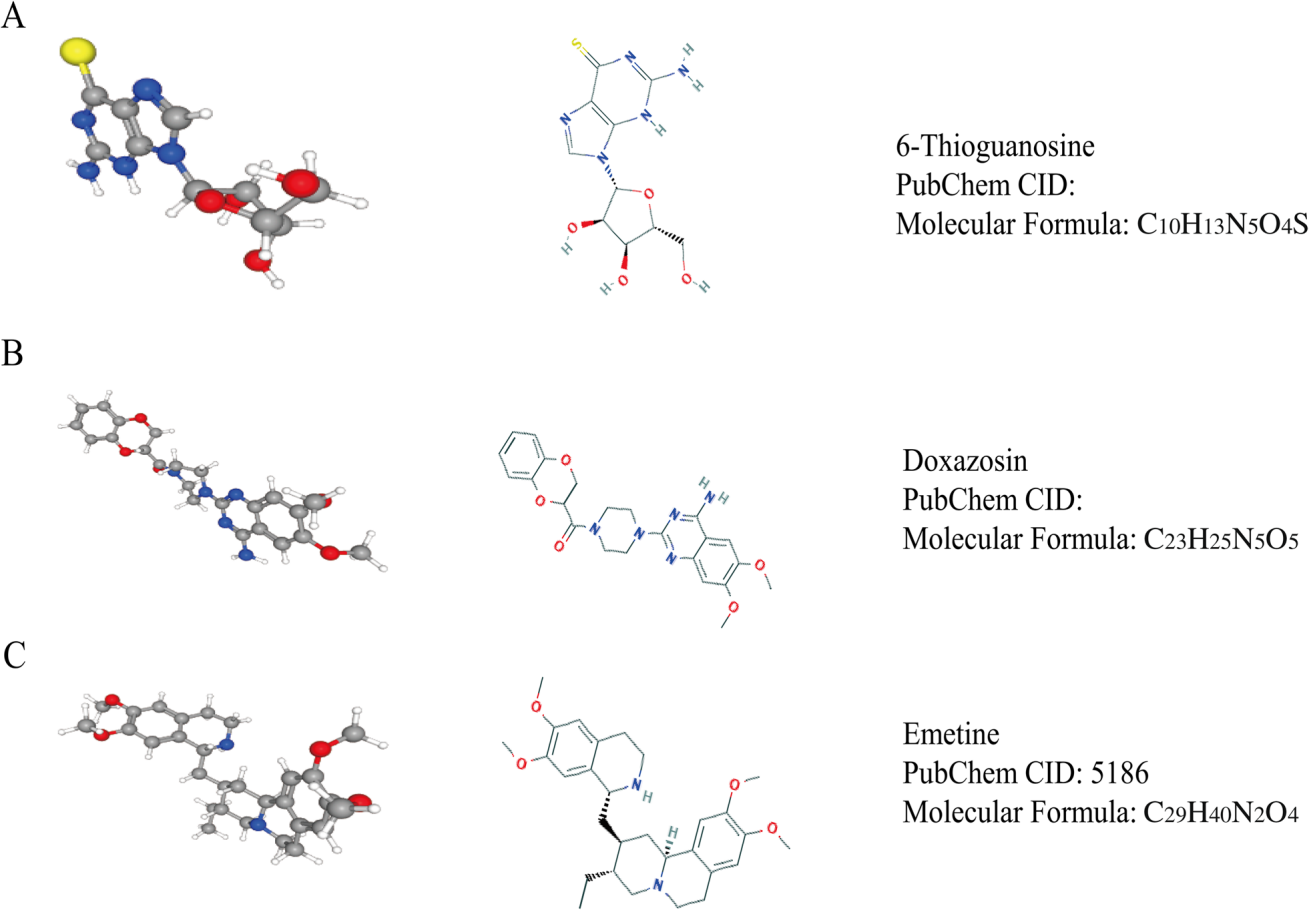
2D and 3D structures of anti-glioma micromolecule drugs targeting *GINS4*. **A** 6-Thioguanosine; **B** doxazosin; **C** emetine

To provide more drugs for the treatment of glioma. We performed CMap analysis based on CGGA RNA-seq data and found three small-molecule potential drugs for the treatment of glioma (6-Thioguanosine, Doxazosin, and Emetine). The corresponding parameters are shown in Table 3, and Fig. 9 shows their two-dimensional and three-dimensional structures.

**Table 3:** Results of CMap analysis

CMap name	Mean	N	Enrichment	p-value
6-Thioguanosine	− 0.718	4	− 0.891	0.00028
Doxazosin	− 0.729	4	− 0.853	0.00092
Emetine	− 0.533	4	− 0.737	0.00951

### Correlation between GINS4 and the immune infiltration of glioma

**Fig. 10:**
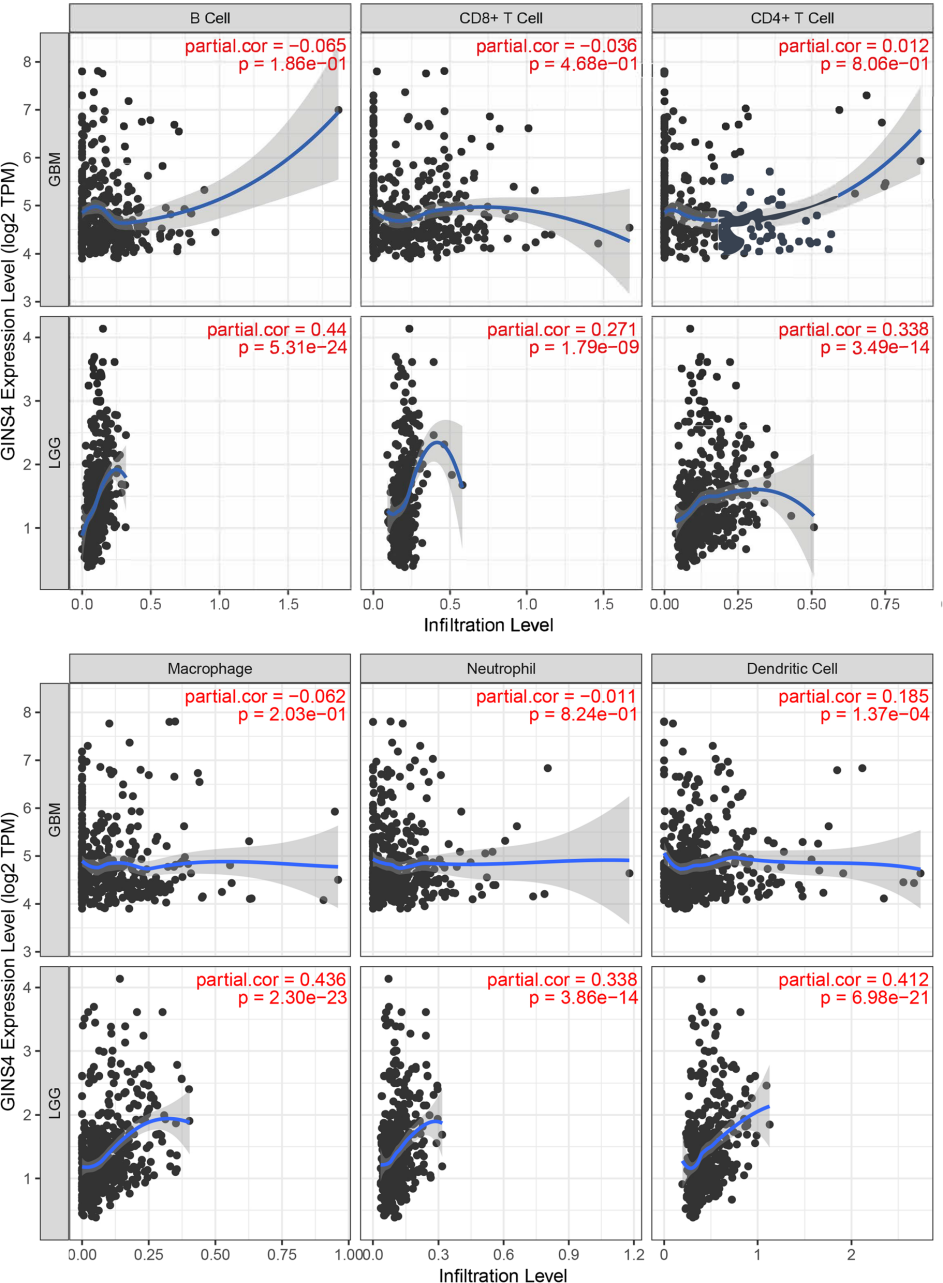
The expression of *GINS4* was associated with immune infiltrating cells

Tumor immune infiltration is a vital factor in neoplasm progress and significantly affects the survival rate of tumor patients (Zhang et al. 2019a). We used the TIMER database to analyze the relationship between mRNA expression of *GINS4* and immune cell infiltration. The results showed that the expression level of *GINS4* was positively correlated with the infiltration levels of B cells (partial cor = 0.44), CD8^+^ T cells (partial cor = 0.271), CD4^+^ T cells (partial cor = 0.338), Macrophages (partial cor = 0.436), Neutrophils (partial cor = 0.338) and Dendritic cells (partial cor = 0.412) in low-grade glioma (LGG) tissues (p < 0.05). In glioblastoma (GBM), the expression level of *GINS4* was positively connected with the Dendritic cell infiltration level (partial cor = 0.185) (p < 0.05). This suggests that *GINS4* may be a potential factor affecting the immune microenvironment of glioma (Fig. 10). In addition, we also explored the relationship between *GINS4* and the Immune checkpoint (CD274, PDCD1, and PDCD1LG2). As shown in Additional file 6: Fig. S2, the *GINS4* expression level is positively correlated with the expression levels of CD274, PDCD1, and PDCD1LG2 in LGG. Collectively, the above results suggest that the expression level of *GINS4* is positively correlated with the level of tumor immune cell infiltration.

## Discussion

The survival rate of glioma patients is poor due to the high morbidity, high recurrence rate, and high mortality of glioma. In recent years, the survival rate of glioma patients has been improved to a certain extent with insight into the pathogenesis of glioma, but there is still a lot of room for improvement. Understanding the specific mechanism of the occurrence and development of glioma from the molecular biology level is of great significance for improving the clinical prognosis of patients. *GINS4*, as a member of the GINS protein family, its act a pivotal part in the initiation of DNA replication and cell cycle (Bermudez et al. 2011; MacNeill 2010). Accumulating evidence has demonstrated that *GINS4* acts as an oncogene to promote the tumorigenesis of various cancer (Yang et al. 2019; Rong et al. 2020; Bu et al. 2020). Nevertheless, a possible role for *GINS4* in the oncogenesis of glioma has not been reported to date.

In this study, we verified that *GINS4* was a prognostic factor in glioma for the first time. In the first place, the data of GEPIA, GEO and HPA database indicated that *GINS4* was upregulated in glioma, and the result of RT-qPCR and IHC staining also proved that the expression of *GINS4* was significantly increased both in glioma cell and tissue. Next, we further analyzed the relationship between the clinicopathological characteristics of glioma patients and found that the expression of *GINS4* was positively correlated with WHO grade, IDH mutation, 1p19 codeletion, PRS_type, and histological subtypes of glioma patients. Once again, the result of Kaplan–Meier survival analysis, ROC curve, univariate and multivariate analyses, and Meta-analysis was confirmed that *GINS4* could be used as an independent prognostic factor to predict poor prognosis in patients with glioma. Finally, our results were consistent with a previous report where to confirm the expression of *GINS4* was elevated both in colorectal cancer (CRC) cell line and tissue, and upregulated expression of *GINS4* can lead to poor prognosis of CRC patients. Hence, our finding suggested that *GINS4* can be used as a potential prognostic factor that affects the prognosis of patients with glioma. However, the mechanism that *GINS4* led to an unfavorable prognosis of glioma needs to be further elucidated.

Subsequently, to explore how *GINS4* takes part in the malignant progress of glioma. We conducted GSEA analysis base on CGGA RNA-seq and TCGA RNA-seq data and found that *GINS4* was significantly enriched in the Notch signaling pathway, pathway in cancer, and JAK-STAT pathway. A growing body of evidence now suggests that those signaling pathways play a critical role in the progress of glioma tumorigenesis and development (Krishna et al. 2019; Yi et al. 2019; Bose et al. 2020; Yang et al. 2019; Zhang et al. 2019b). For instance, the Notch signaling pathway plays an important role in cell differentiation, survival, proliferation, stem cell renewal, and determining cell fate during development and morphogenesis (Krishna et al. 2019). Meantime, the abnormal activation of Janus kinase (JAK)/signal transducer and activator of transcription (STAT) signaling pathway can promote carcinogenesis (Bose et al. 2020; Yang et al. 2019; Zhang et al. 2019b). Moreover, GSEA is widely used to reveal the potential molecular mechanism of a special gene in the pathological process of the disease, and its reliability is high. Compared with traditional analysis methods, GSEA has the advantage of a large sample size, which can avoid the inherent deviation of experimental results caused by artificially setting thresholds (Subramanian et al. 2005). Several reports have shown that the reliability of GSEA. For instance, Liu et al. based on GSEA analysis proved that the upregulation of *HOXA2* could be modulating the focal adhesion and JAK-STAT signaling pathways in glioma (Liu et al. 2020). Collectively, it is reasonable to believe that *GINS4* affects the prognosis of glioma through the above-mentioned cancer-related signaling pathways.

Similar to other cancers, the tumorigenesis of glioma is a multi-step, multi-genes, and complicated progress. Therefore, we tried to discover more potential genes which take part in the occurrence and development of gliomas through co-expression analysis. Prior studies had confirmed that some of the *GINS4* positive correlation genes play a vital role in the pathology of glioma. For instance, Xiaozhi Li et al. suggest ed that *RFWD3* has a good ability to predict the overall survival of patients with glioma (Li and Meng 2021). *BUB1*, as a novel therapeutic target for glioma can promote the proliferation and radio-resistance ability of glioma (Yu et al. 2019). Many researchers have demonstrated that *CDK2* acts a key role in the proliferation, migration, and invasion of human glioma (Guo et al. 2018; Gao et al. 2017). In addition, *CENPL*, *ESCO2*, and *CLSPN* also serve important roles in tumor progression (Yin et al. 2021; Chen et al. 2018; Cai et al. 2021). In terms of negatively correlated *GINS4* genes, previous studies have confirmed that they may be a novel tumor suppressor in cancer. For instance, *FBXW4* is lost and downregulated in many cancers, which maybe lead to a poor prognosis (Lockwood et al. 2013). Based on the results of previous studies and this study, we have more reason to believe that *GINS4* plays an indispensable role in glioma, and it may play a role in the malignant process of glioma together with *RFWD3*, *BUB1*, *CDK2*, *CENPL*, *ESCO2*, and *CLSPN*. Thus, the results of co-expression analysis of *GINS4* bring new insight to gliomagenesis.

Once more, to use the carcinogenic effect of *GINS4* in gliomas for clinical treatment of gliomas. We explore small molecular drugs for the chemical treatment of glioma by using CMap online analysis tool and found three potential drugs that have potential value in clinical application. While these drugs have not been used in the treatment of glioma, a great of prior studies have proven that their anti-cancer properties in different cancer types (Vethe et al. 2008; Gaelzer et al. 2016; Wu et al. 2019; Sun et al. 2019; Alam et al. 2020). For instance, 6-thioguanosine can change the basal activity of 5′-monophosphate (IMP) in human leukemia cells (Vethe et al. 2008). In recent years, the reapplication of traditional medicine has received continuous attention, and satisfactory results have been achieved in basic research and clinical application. Aspirin, as a classic example of drug repurposing, has a better effect on relieving mild or moderate pain. It is widely used clinically to prevent a transient ischemic attack, myocardial infarction, artificial heart valves, and venous fistulas or another thrombosis after surgery (McFadyen et al. 2018). Taken together, it is reasonable to believe that the above-mentioned three drugs have new clinical application value for the treatment of glioma by targeting *GINS4*.

In recent years, the clinical application of cancer immunotherapy has made significant progress, and it has become another effective treatment method for cancer following surgery, radiotherapy, chemotherapy, and targeted therapy (Ge et al. 2017). As the main component of the tumor microenvironment, immune infiltration plays a central role in tumorigenesis and development and has been proven to contribute to tumor progression and immunotherapy response (Aaes and Vandenabeele 2021). However, there weren’t studies that have reported the correlation between *GINS4* and immune infiltration in gliomas. In the present study, we found that the expression of *GINS4* was positively correlated with Dendritic cells in LGG and GBM. A previous study proved that Nrf2 was facilitating the immune escape of glioma cells by suppressing Dendritic cell function (Wang et al. 2017). This suggests that *GINS4* may exert its oncogene effect by affecting the function of Dendritic cells. In addition, we explore the relationship between *GINS4* expression and immune checkpoints, including CD274, PDCD1, and PDCD1LG2, and revealed that there significant positive co-expression correlations between immune checkpoints and *GINS4*. Immune checkpoints are a class of immunosuppressive molecules that can regulate T cell activity through a series of pathways such as co-suppression or costimulation signals to improve the anti-tumor immune response (Dyck and Mills 2017). There is increasing evidence that immune checkpoints that can play a role in glioma progression (Lombardo et al. 2020). Hence, the above results demonstrated that *GINS4* was implicated in the tumor immune microenvironment mainly through the regulation of Dendritic cell function and Immune checkpoint (CD274, PDCD1, and PDCD1LG2), and *GINS4* may affect the survival rate of patients by affecting the tumor immune microenvironment in glioma.

However, we should notice that there were still some inevitable limitations in our study. Firstly, due to the limitations of the CGGA and TCGA databases, some clinical features of glioma patients are incomplete, such as the lack of data on glioma tissue subtypes. In addition, the lack of data of GINS4 in non-invasive tissues in the database limits us comprehend the status in non-invasive tissues, which requires further exploration in future research. Nevertheless, it is encouraging to note that we have analyzed the correlation between *GINS4* and glioma at multiple levels and multiple databases to ensure the comprehensiveness and reliability of our research, and our study results are noteworthy in the field of identifying promising prognostic biomarkers for glioma.

## Conclusion

In conclusion, this study revealed that *GINS4*, as a promising prognostic biomarker, was upregulated in glioma and positively related to the clinical character and survival of glioma. The upregulation of *GINS4* predicts an unfavorable prognosis in glioma. Moreover, *GINS4* potentially promotes the malignant processes of glioma by participating JAK-STAT pathway, etc., other cancer-related pathways, and regulating the immune microenvironment. Hence, *GINS4* act as a novel biomarker plays an important role in the prognosis of glioma and has potential clinical application for improving the prognosis status of glioma.

## Supplementary Information

**Additional file 1: Table S1.** Sequences of primers used for RT-qPCR.

**Additional file 2: Table S2.** Characteristics ofpatients with gliomabased on CGGA RNA-seq data.

**Additional file 3: Table S3.** Characteristics ofpatients with gliomabased on CGGA microarraydata.

**Additional file 4: Table S4.** Characteristics ofpatients with gliomabased on TCGA RNA-seq data.

**Additional file 5: Fig. S1.** Representative IHC imagesof *GINS4* protein expression in glioma tissues and corresponding normalbrain tissues base on the HPA database. A, D. IHC images of *GINS4 *protein in normal brain tissue; B, E. IHC images of *GINS4* protein in lowgrade glioma; C, F. IHC images of *GINS4* protein in high grade glioma.

**Additional file 6: Fig. S2.** The relationship between *GINS4 *expression and Immune checkpoint (CD274, PDCD1, and PDCD1LG2).

## Data Availability

The datasets used and analyzed during the current study are available from the corresponding author on reasonable request.

## Abbreviations

CGGA: Chinese Glioma Genome Atlas
TCGA: The Cancer Genome Atlas
GEO: Gene Expression Omnibus
GEPIA: Gene Expression Profiling Interactive Analysis
HPA: Human Protein Atls
TIMER: Tumor Immune Estimation Resource
GSEA: Gene set enrichment analysis
CMap: Connectivity map
MGMT: O-6-methylguanine-DNA methyltransferase
EGFR: Epidermal growth factor receptor
TP53: Tumor protein p53
IDH mutation: Isocitrate dehydrogenase
GBM: Glioblastoma multiforme
LGG: Low-grade gliomas
IHC: Immunohistochemical
siRNA: Small interfering RNA
HA: Human astrocyte
KEGG: Kyoto Encyclopedia of Genes and Genomes
NOM: Nominal
FDR: False discovery rate
OS: Overall survival
HR: Hazard ratio
CI: Confidence interval
TIICs: Tumor-infiltrating immune cells
NT: Non-tumor
ROC: Receiver operating characteristic
RT-qPCR: Real-time quantitative polymerase chain reaction
AUC: Area under the receiver operating characteristic
CRC: Colorectal cancer
