# Supplementary material for: Overexpression of GINS4 is associated with poor prognosis and survival in glioma patients
Source: Mol Med. 2021 Sep 23;27:117. doi: 10.1186/s10020-021-00378-0 (PMC8461916; doi:10.1186/s10020-021-00378-0)
Supplement: Supplementary file 1 — Additional file 1: Table S1. Sequences of primers used for RT-qPCR. [file 10020_2021_378_MOESM1_ESM.doc]

**Table S1.** Sequences of primers used for RT-qPCR.

| Gene | Primer sequence (5′-3′) |
| --- | --- |
| CYP17A1-AS1(F) | TCCAGTCCCTTCCCAGTAGT |
| CYP17A1-AS1(R) | CTCTGGCAGCCAAGCAGTAG |
| MYH7B(F) | TCGCTTCGACTTACTGGAGGA |
| MYH7B(R) | GGAGGCCGTATAGACTGGGA |
| CCNI2(F) | CGCCTTCGAGGAAGTCGTG |
| CCNI2(R) | CCAGGTTAAAAGTGGACTGGG |
| FBXW4(F) | GTGCTTACACACCATCCAGAC |
| FBXW4(R) | ACTGTTGAGGTCCCAGATTCT |
| RASL10A(F) | AGTTCCTGTTCGGTGACTACC |
| RASL10A(R) | CAAGCTCCAGTCCTTAGCGTC |
| NOXA1(F) | TGCTACACAATGTGGCGTC |
| NOXA1(R) | ACTTGGACATGGCCTCCCTTA |
| MATK(F) | GATGAGGCCGTGTTCTTCTG |
| MATK(R) | TGCTCCCAATGTCAAATGCTG |
| C1QTNF4(F) | CGGCTACCTAGTCTACGCC |
| C1QTNF4(R) | AAGTCGCCGCCAATGTTGA |
| ANKRD24(F) | GAGGCGTCATCTCAGAACTCT |
| ANKRD24(R) | GATCATCCGGCATGGGAATG |
| LINC00634(F) | TTCTTCGTGAAGTCCCCAGC |
| LINC00634(R) | AGCTGACATCCCTCCAAACG |
| CENPL(F) | CACCAGAGTCAACTCCTAGTGC |
| CENPL(R) | TCTGCTTCCTGACCGATTCTAA |
| ESCO2(F) | CACTGGGACGCACCCAAAA |
| ESCO2(R) | CACTTGCCTTGTCGCAAAAG |
| PRR11(F) | AAAGATGGACCCATGCAGATAAC |
| PRR11(R) | TGCTTTCGGCGATGGTATAAG |
| MCM4(F) | GACGTAGAGGCGAGGATTCC |
| MCM4(R) | GCTGGGAGTGCCGTATGTC |
| NDE1(F) | CTGCAACAAATTGAAACCAGGAA |
| NDE1(R) | CTGCCGGTAGCCTTCAGAG |
| CLSPN(F) | TGGAGAGTGGGGTCCATTCAT |
| CLSPN(R) | CCGGGGTTTACGTTTGAAGAAA |
| CDK2(F) | GTACCTCCCCTGGATGAAGAT |
| CDK2(R) | CGAAATCCGCTTGTTAGGGTC |
| BUB1(F) | ACAATCAACGGAGAAAGCATGA |
| BUB1(R) | CTCCACCACCTGATGCAACT |
| MCM8(F) | AATGGAGAGTATAGAGGCAGAGG |
| MCM8(R) | CAGAAGTACGTTTTCCTGTGGT |
| RFWD3(F) | AACATAGGCAGGGATCTGATGG |
| RFWD3(R) | AATGGTGCTCTCAACCTGGC |
| GINS4(F) | TCTACGTCCTCATGCCTCCA |
| GINS4(R) | GATGTAAAGTGCTCCGTGCAG  GCTCCGTGCAG |
| GAPDH(F) | CAAGGTCATCCATGACAACTTTG |
| GAPDH(R) | GTCCACCACCCTGTTGCTGTAG |
