# Supplementary material for: Overexpression of GINS4 is associated with poor prognosis and survival in glioma patients
Source: Mol Med. 2021 Sep 23;27:117. doi: 10.1186/s10020-021-00378-0 (PMC8461916; doi:10.1186/s10020-021-00378-0)
Supplement: Supplementary file 4 — Additional file 4: Table S4. Characteristics ofpatients with gliomabased on TCGA RNA-seq data. [file 10020_2021_378_MOESM4_ESM.docx]

Table S4. Characteristics of patients with glioma based on TCGA RNA-seq data.

| Characteristics |  | Number of cases | Percentages(%) |
| --- | --- | --- | --- |
| Gender | Male | 377 | 57.73 |
|  | Female | 276 | 42.27 |
| Age | <=51 | 394 | 60.34 |
|  | >51 | 259 | 39.66 |
| Grade | WHO II | 238 | 36.45 |
|  | WHO III | 256 | 39.20 |
|  | WHO IV | 159 | 24.35 |
