# Supplementary figures and images for: Overexpression of GINS4 is associated with poor prognosis and survival in glioma patients
Source: Mol Med. 2021 Sep 23;27:117. doi: 10.1186/s10020-021-00378-0 (PMC8461916; doi:10.1186/s10020-021-00378-0)

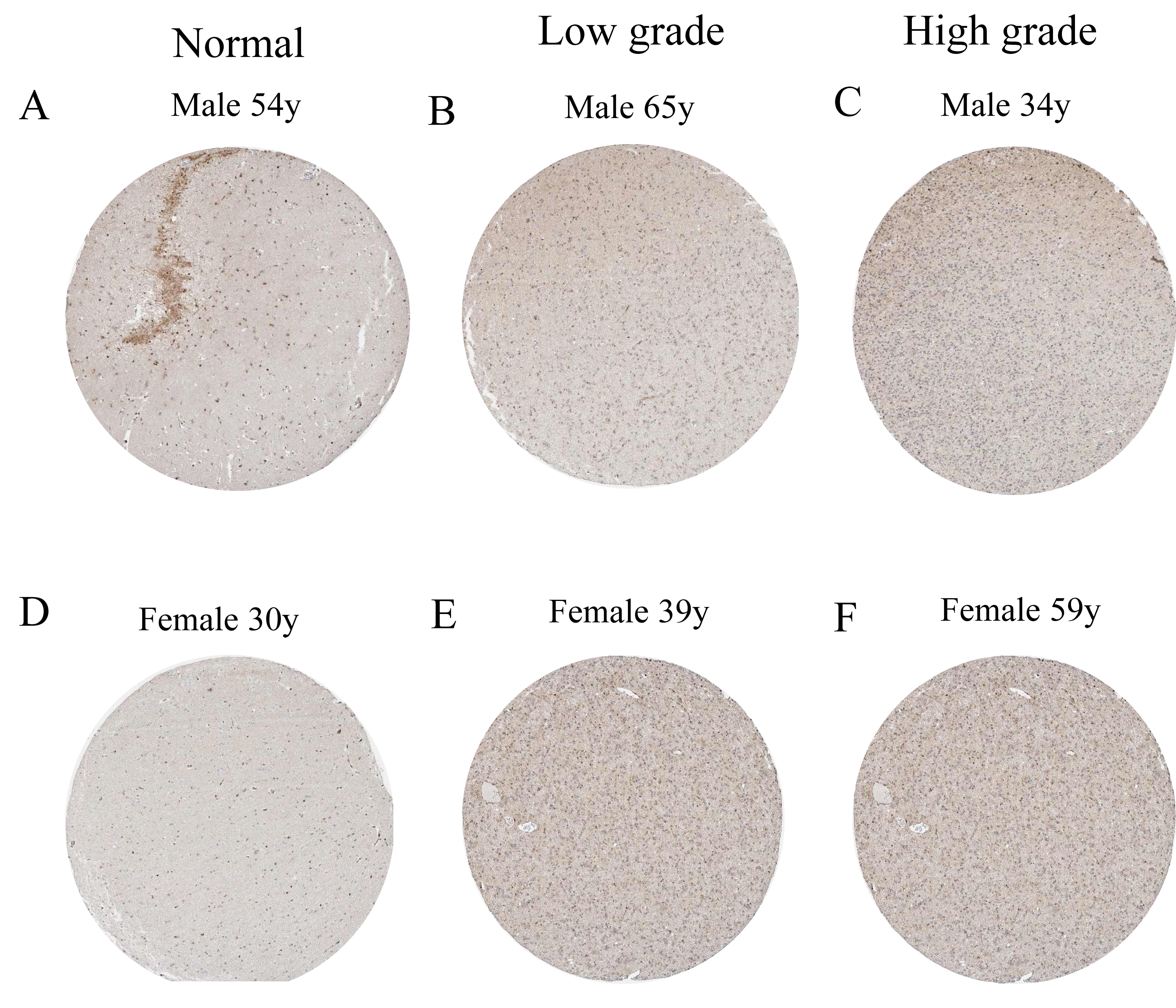

Supplement: Supplementary file 5 — Additional file 5: Fig. S1. Representative IHC imagesof GINS4 protein expression in glioma tissues and corresponding normalbrain tissues base on the HPA database. A, D. IHC images of GINS4 protein in normal brain tissue; B, E. IHC images of GINS4 protein in lowgrade glioma; C, F. IHC images of GINS4 protein in high grade glioma. [file 10020_2021_378_MOESM5_ESM.tif]

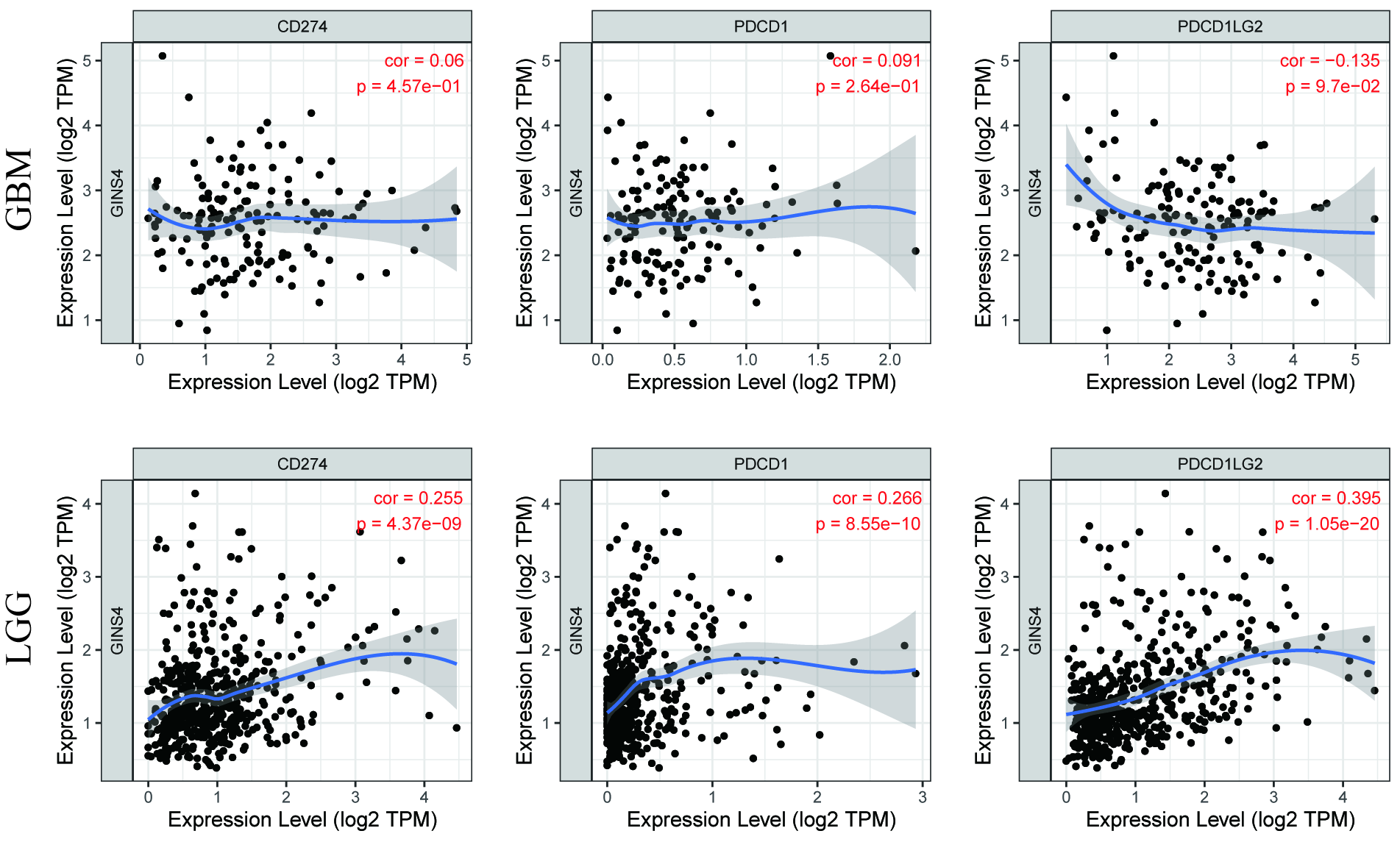

Supplement: Supplementary file 6 — Additional file 6: Fig. S2. The relationship between GINS4 expression and Immune checkpoint (CD274, PDCD1, and PDCD1LG2). [file 10020_2021_378_MOESM6_ESM.tif]
